# TSG101 promotes the proliferation, migration and invasion of hepatocellular carcinoma cells by regulating the PEG10

**DOI:** 10.1111/jcmm.13878

**Published:** 2018-11-18

**Authors:** Zhiyi Liu, Zilu Tian, Kuan Cao, Bin Zhang, Quan Wen, Xinyu Zhou, Weibin Yang, Tao Wang, Hengliang Shi, Renhao Wang

**Affiliations:** ^1^ Institute of Digestive Diseases Xuzhou Medical University Xuzhou Jiangsu China; ^2^ The Graduate School Xuzhou Medical University Xuzhou Jiangsu China; ^3^ Department of General Surgery Affiliated Hospital of Xuzhou Medical University Xuzhou Jiangsu China

**Keywords:** HCC, MMPs, p21, p53, PEG10, TSG101

## Abstract

The tumour susceptibility gene 101 (TSG101) is reported to play important roles in the development and progression of several human cancers. However, its potential roles and underlined mechanisms in human hepatocellular carcinoma (HCC) are still needed to be further clarified. In the present study, we reported that knock down of TSG101 suppressed the proliferation, migration and invasion of HCC cells, while overexpression of TSG101 facilitated them. Molecularly, the results revealed that knock down of TSG101 significantly decreased the cell cycle related regulatory factor p53 and p21. In another point, knock down of TSG101 also obviously decreased the level of metallopeptidase inhibitor TIMP1 (Tissue inhibitors of metalloproteinases 1), which results in inhibition of MMP2, MMP7 and MMP9. In contrast, overexpression of TSG101 had opposite effects. The iTRAQ proteomics analysis identified that oncogenic protein PEG10 (Paternally expressed gene 10) might be a potential downstream target of TSG101. Further investigation showed that TSG101 interacted with PEG10 and protected it from proteasomal degradation thereby regulating the expression of p53, p21 and MMPs. Finally, we found that both TSG101 and PEG10 proteins are up‐regulated and presented a direct correlation in HCC patients. In conclusion, these results suggest that TSG101 is up‐regulated in human HCC patients, which may accelerate the proliferation, migration and invasion of HCC cells through regulating PEG10.

## 1 INTRODUCTION

Hepatocellular carcinoma is one of the leading causes of cancer death worldwide. Despite the great advances in diagnosis and treatment in the past decade, the outcome of patients with HCC is still very poor.^1^, ^2^, ^3^, ^4^ Curing this intractable disease therefore requires a profound understanding of the mechanisms underlying its pathogenesis, which is critical to identify specific molecular targets that could be served as a treatment for HCC.

TSG101, an important member of the ESCRT‐I (endosomal complexes required for transport), has been shown to be involved in many cellular processes, such as endosomal trafficking, ubiquitination, virus budding and cell survival.^5^, ^6^, ^7^, ^8^, ^9^ Biochemical studies have identified that TSG101 can regulate the protein ubiquitination by interacting with many ubiquitin ligases, such as Tal,^10^, ^11^ MGRN1,^12^, ^13^ MDM2.^14^, ^15^ Recently, it is reported that TSG101 plays important roles in the development and progression of human cancers. For example, the expression of TSG101 is frequently up‐regulated in human ovarian cancer, colorectal carcinoma, papillary thyroid carcinoma, gastrointestinal tumour, and gallbladder cancer,^16^, ^17^, ^18^, ^19^, ^20^ thus it could be served as a biomarker in several human cancers. Overexpression of TSG101 in cancers is found to activate multiple important pathways, including PI3K/Akt and MAPK/ERK pathways.^21^ In addition, TSG101 is reported to play a role in promoting HCC cell growth,^22^ however, the underlined mechanism and the potential role of TSG101 in human HCC migration and invasion remains unknown, which should be fully clarified.

PEG10, also known as EDR, HB‐1, Mar2, MEF3L, Mart2, and RGAG3, is expressed not only in brain, kidney, and lung tissues in adults but also in embryonic tissues, such as placenta.^23^ It has been demonstrated that multiple roles have been attributed to PEG10, for example, it involves in cell proliferation,^24^, ^25^ inhibition of cell apoptosis^26^ and promotion of migration and invasion.^27^ In addition, some studies have indicated that PEG10 is frequently overexpressed in multiple malignancies, such as lung cancer, hepatocellular carcinoma, B‐cell chronic lymphocytic leukemia and pancreatic cancer.^27^, ^28^, ^29^, ^30^ The overexpression of PEG10 is significantly associated with the proliferation, migration and metastasis of such malignancies.

In this study, we observed the expression of TSG101 protein in HCC tissues and investigated the roles of TSG101 in the proliferation, migration and invasion of HCC cells. We provided evidence that TSG101 promotes the proliferation, migration and invasion of HCC cells through regulating the expression of PEG10.

## 2 MATERIALS AND METHODS

### 2.1 Antibodies

TSG101, PEG10, TIMP1, MMP2, MMP7, MMP9 and FLAG antibodies were bought from Abcam (Cambridge, UK). Antibodies against p53, p21 and β‐actin were purchased from Cell Signaling Technology (Danvers, MA, USA).

### 2.2 Tissue samples

Fourteen specimens of human HCC tissues and fourteen specimens of normal liver tissues were collected at the Affiliated Hospital of Xuzhou Medical University (Xuzhou, China). Surgically removed tissues were sampled for histological diagnosis, and the remaining tissues were immediately frozen in liquid nitrogen and stored at −80°C in the fridge. All specimens have been confirmed by the pathological diagnosis. Written informed consent was acquired from each patient, and the study was permitted by the Research Ethics Committee of Xuzhou Medical University.

### 2.3 Cell culture

HCC cell lines HepG2 and SMMC‐7721 and human embryonic kidney cell line 293T (HEK293T) were purchased from the cell bank of Shanghai Institutes of Chinese Academy of Sciences. Cells were cultured in Dulbecco's modified Eagle's medium (Invitrogen, Carlsbad, CA, USA) supplemented with 10% foetal bovine serum (TransGen, Beijing, China) in a 5% CO_2_ incubator at 37°C.

### 2.4 Constructs and production of the lentivirus

For silencing of TSG101, three shRNA duplexes were designed as follows:

shTSG101#1‐F:

GATCGCAGTTCCAGGGAACTAATTTCAAGAGAATTAGTTCCCTGGAACTGCTTTTTTG

shTSG101#1‐R:

AATTCAAAAAAGCAGTTCCAGGGAACTAATTCTCTTGAAATTAGTTCCCTGGAACTGC

shTSG101#2‐F:

GATCGCTTATTCAGGTCATGATTTTCAAGAGAAATCATGACCTGAATAAGCTTTTTTG

shTSG101#2‐R:

AATTCAAAAAAGCTTATTCAGGTCATGATTTCTCTTGAAAATCATGACCTGAATAAGC

shTSG101#3‐F:

GATCGGATGTCTTCCTGAAGCATTTCAAGAGAATGCTTCAGGAAGACATCCTTTTTTG

shTSG101#3‐R:

AATTCAAAAAAGGATGTCTTCCTGAAGCATTCTCTTGAAATGCTTCAGGAAGACATCC

Control‐F:

GATCTTCTCCGAACGTGTCACGTTTCAAGAGAACGTGACACGTTCGGAGAATTTTTTG

Control‐R:

AATTCAAAAAATTCTCCGAACGTGTCACGTTCTCTTGAAACGTGACACGTTCGGAGAA

The shRNA oligomers and nontargeting oligomers (control) were annealed and then subcloned into the pLV‐shRNA vector by the *BamH* I and *EcoR* I cloning sites. Cell transfection was performed with a PolyJet (SignaGen, Gaithersburg, MD, USA) as described in the manufacturer's protocol. Cell transfection was carried out by PolyJet (SignaGen, Gaithersburg, MD, USA) according to the manufacturer's instructions. The lentiviruses were produced by co‐transfecting the core plasmid and the packaging plasmids in 293T cells.

### 2.5 Establishment of the stable cell lines

The establishment of stable cell lines was performed as we previously described.^31^, ^32^ For stably silencing of TSG101, HepG2 and SMMC‐7721 cells were infected by control and shTSG101#3 viruses, respectively. Forty‐eight hours after infection, the cells were continuously cultured in the medium containing 2.5 μg/mL puromycin (Sigma, St. Louis, MO, USA). The surviving cells were cultured into cell lines stably expressing control and shTSG101#3.

### 2.6 Transient overexpression of TSG101

The TSG101 construct was generated by cloning the human TSG101 cDNA into the expression vector p3XFLAG‐CMV‐14 at the *Hind* III and *Kpn* I restriction sites. Transfection of TSG101 was performed using the PolyJet transfection reagent according to the manufacturer's instructions.

### 2.7 EdU assay

The effect of TSG101 on the proliferation of HepG2 and SMMC‐7721 cells was measured by 5‐ethynyl‐20‐deoxyuridine (EdU) incorporation assay using EdU assay kit (Ribobio, Guangzhou, China) according to the manufacture's protocol. Briefly, the cells were cultivated in 96‐well plates at 4 × 10^3^ cells/well. Twenty hours after culture, the cells were applied to 50 μmol/L of 5‐ethynyl‐20‐deoxyuridine (EdU; Ribobio, Guangzhou, China) and incubated for 2 hours at 37°C. The cells were washed with PBS and fixed with 4% paraformaldehyde for 20 minutes, and then permeabilised with 0.5% Triton X‐100 for another 20 minutes. Afterwards, the cells were washed five times with PBS and incubated with 100 μL of 1 × Apollo^®^ reaction cocktail for 30 minutes at room temperature. Finally, the nuclei of the cells were dyed with 100 μL of Hoechst 33342 (5 μg/mL) for 20 minutes and visualised with a fluorescent microscopy (IX71; Olympus, Tokyo, Japan).

### 2.8 Cell viability assay

The cell viability was assessed via cell counting kit‐8 assay (CCK‐8, Dojindo, Janpa). Cells were plated at 3000 per well in triplicate in 96‐well plates. At the designated time point, 10 μL of CCK‐8 reagent was applied into the medium. After reaction for 4 hours at 37°C, the absorbances at 450 nm were determined by a SynergyMx Multi‐Mode Microplate Reader (Biotek, Winooski, VT). The cell viability was calculated according to the absorbances.

### 2.9 Plate colony formation

5 mL of cell suspension containing 400 cells was inoculated into a diameter 60 mm dish for continuous culture until the visible clones appeared. Then, the cells were fixed with methanol and stained with 0.05% crystal violet solution. After washing twice with PBS, the plates were photographed using a digital camera. Positive colony formation, defined as colonies with more than 50 cells, was confirmed by manual counting.

### 2.10 Wound healing assay

The migration function of cells was evaluated using wound healing assay. The cells were seeded in 6‐well plates. Then, the scratches were developed in the middle of the wells with a pipette tip. The cells were washed with PBS to remove the debris and incubated in serum‐free media for 48 hours. At the designated time, five randomly selected fields were acquired under an inverted microscope (Olympus). The number of cells across the wound was normalised to the control group.

### 2.11 Transwell invasion and migration assays

Transwell assays were performed with a polycarbonate filter membrane with a diameter of 6.5 mm and pore size of 8 μm (Invitrogen) according to the manufacturer's protocol. To assess invasion ability, the filters were pre‐coated with 10 μg of Matrigel (BD). The cell suspension (1 × 10^4^) in serum‐free culture medium containing 10 μM CDK2 inhibitor (K03861) was added into the inserts, and each insert was placed in the lower chamber filled with culture media containing 10% foetal bovine serum as a chemoattractant. After 48 hours of incubation at 37°C, the non‐invasive cells were removed from the upper chamber by wiping with cotton‐tipped swabs. Then, the filters were fixed with methanol for 15 min and stained with a 0.1% crystal violet solution for 10 min. Five fields of adherent cells in each well were randomly photographed with an inverted microscope and counted. The same experimental design was used for migration experiments except that filters were not pre‐coated with Matrigel.

### 2.12 Quantitative iTRAQ‐based proteomic analysis

Quantitative iTRAQ‐based proteomic analysis was performed by CapitalBio Technology Co. Ltd (Beijing, China). Total protein was extracted from HepG2‐Control and HepG2‐shTSG101#3 cells. 100 μg of each protein was denatured in 8 mol/L urea in 50 mmol/L NH_4_HCO_3_ pH 7.4 and alkylated with 10 mmol/L iodoacetamide for 1 hour at 37°C. Then each sample was diluted 10‐fold with 25 mmol/L NH_4_HCO_3_ and digested with trypsin at a ratio of 1:100 (trypsin/substrate) for 6 hours at 37°C. A 25 lg aliquot of digested peptides for each sample was subjected to eight‐plex iTRAQ labelling according to the manufacturer's instructions. Peptides from each iTRAQ experiment were subjected to capillary liquid chromatography‐tandem mass spectrometry (LC‐MS/MS) using a Q Exactive Hybrid Quadrupole‐Orbitrap Mass Spectrometer (Thermo Fisher Scientific, Waltham, MA, USA). The quantitative analysis was conducted by calculating the ratios between experimental group and control group. To make the data more credible, the iTRAQ experiment was repeated at three times. The changes were considered significant if the increased or decreased fold change >1.5 and the *P* < 0.05. The original mass spectrum data were searched by database using Mascot 2.2 and Proteome Discoverer 1.4 (Thermo Fisher Scientific, Waltham, MA, USA).

### 2.13 Western blotting

The cells were lysed in RIPA buffer and centrifuged at 12 000 *g* for 10 minutes at 4°C. The supernatants were collected for Western blotting assay. Equal amount of proteins was subjected to 10% SDS‐PAGE and then transferred to 0.45‐μm pore size PVDF membrane (Millipore, Billerica, MA, USA). After blocking with 5% nonfat milk, the membrane was probed with primary antibodies (TSG101, FLAG, PEG10, TIMP1, MMPs, p53, p21 and β‐actin) at 4°C overnight and secondary antibodies at room temperature for 1 hour. Bound antibodies were detected by the ECL Plus western blotting substrate (Thermo Fisher, Waltham, MA, USA) and detected by enhanced chemiluminescence detection system (Thermo Fisher). Band densities were quantified by Image J Software. The relative amount of proteins was determined by normalizing the densitometry value of interest to that of the loading control.

### 2.14 Co‐immunoprecipitation

The cells were transiently transfected with the indicated plasmids. Twenty‐four hours after transfection, the cells were lysed in a Triton‐X‐100‐based lysis buffer (1% Triton‐X100, 150 mMNaCl, 20 mM 4‐(2‐Hydroxyethyl)‐1‐piperazineethanesulfonic acid (HEPES), pH 7.4, 2 mM ethylenediaminetetraaceticacid, 5 mM MgCl2 supplemented with protease inhibitor) for 20 min on ice. The nuclear and cellular debris were cleared by centrifugation. One milligram of total protein was subjected for immunoprecipitation with the indicated antibody. The immunoprecipitates were washed five times in lysis buffer, and proteins were recovered by boiling the beads in an SDS sample buffer and analysed by western blotting.

### 2.15 Immunohistochemistry

Immunohistochemical staining was performed using the protocol supplied by the S‐P immunohistochemistry kit (Zhongshan Goldenbridge Biotech CO., Beijing, China). The sections were fixed with 4% paraformaldehyde and blocked with 10% goat serum. Then, the sections were incubated with TSG101 or PEG10 antibody followed by Biotin‐conjugated goat anti‐rabbit IgG and HRP‐conjugated streptavidin. The reaction was developed by 3, 3′‐diaminobenzidine (DAB) chromogenic reagent (Zhongshan Goldenbridge Biotech CO.). The sections were counterstained with hematoxylin to stain the nucleus and dehydrated by incubation in increasing concentrations of alcohol, followed by 100% xylene. Finally, the cover slips were mounted onto the slides with neutral gum. The photos were collected under an Olympus IX‐71 microscope (Olympus). The images were processed by Image‐Pro‐Plus 6.0 software, and the mean optical density (MOD) represented the expression level of TSG101 and PEG10.

### 2.16 Statistical analysis

The results shown were representative of experiments that repeated at least three times. All the quantitative data was presented as mean ± SEM. Statistical analysis was performed with the SPSS Version 13.0 (SPSS Inc, Chicago, IL, USA). Differences in multiple groups were compared by a one‐way analysis of variance (ANOVA) followed by post hoc test. Differences between two groups were determined by Student's *t* test. *P* < 0.05 were considered statistically significant (*P <* *0.05).

## 3 RESULTS

### 3.1 Knock down of TSG101 inhibits the proliferation, migration and invasion of HCC cells

**Figure 1:**
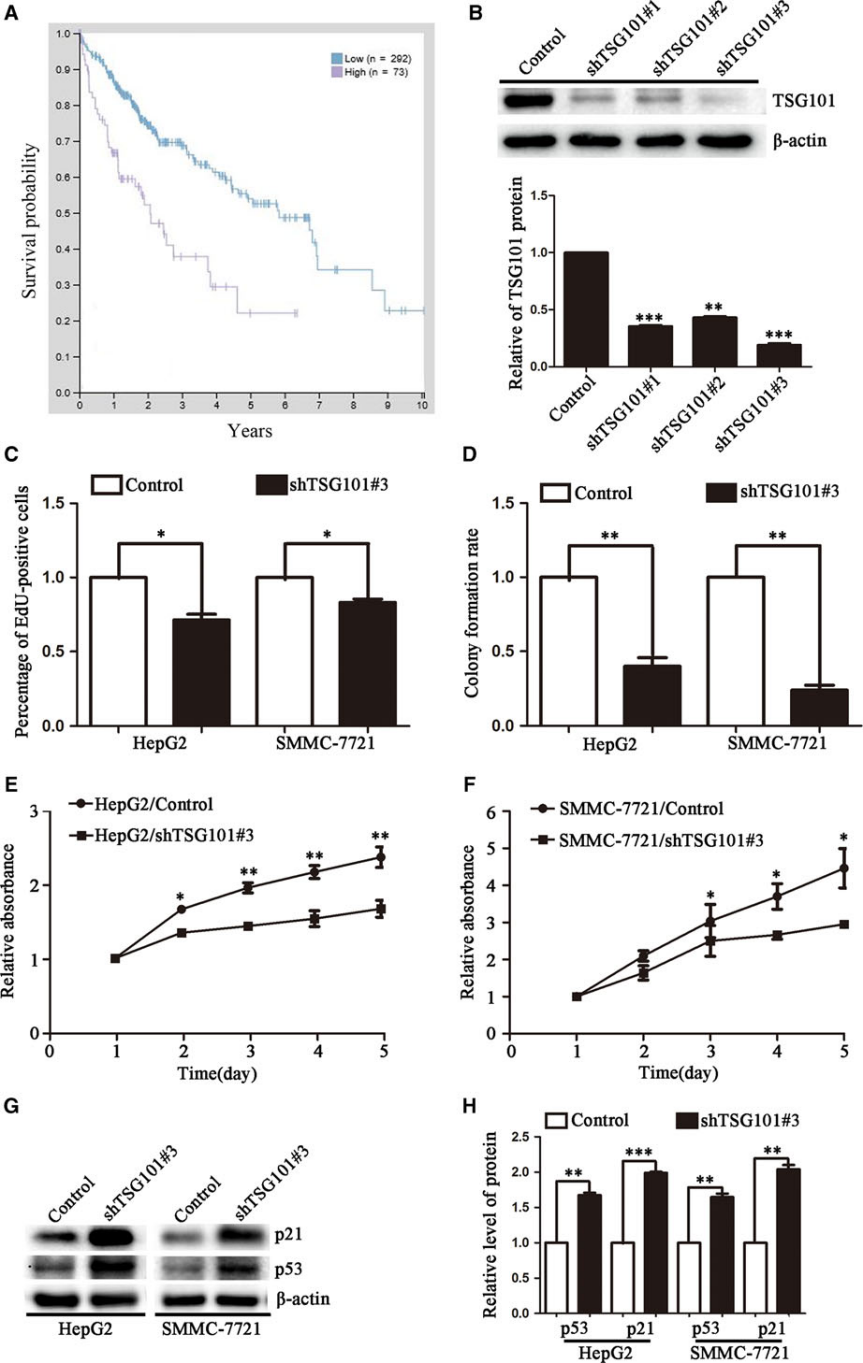
Down‐regulation of TSG101 inhibits HCC cell proliferation. A, TCGA database analysis showed high levels of TSG101 were associated with poor prognosis in HCC patients. B, Representative blots and quantification of shTSG101 sefficiency. C, Quantification of the EdU incorporation assay performed in HepG2 and SMMC‐7721 cells upon knocking down of TSG101. D, Quantification of the colony formation assay with TSG101 silenced HepG2 and SMMC‐7721. E and F, CCK8 assay results. G, Representative bolts and H, quantification to show the protein levels of p53 and p21 in TSG101 silenced HepG2 and SMMC‐7721 cells. *P < 0.05, **P < 0.01, ***P < 0.001

**Figure 2:**
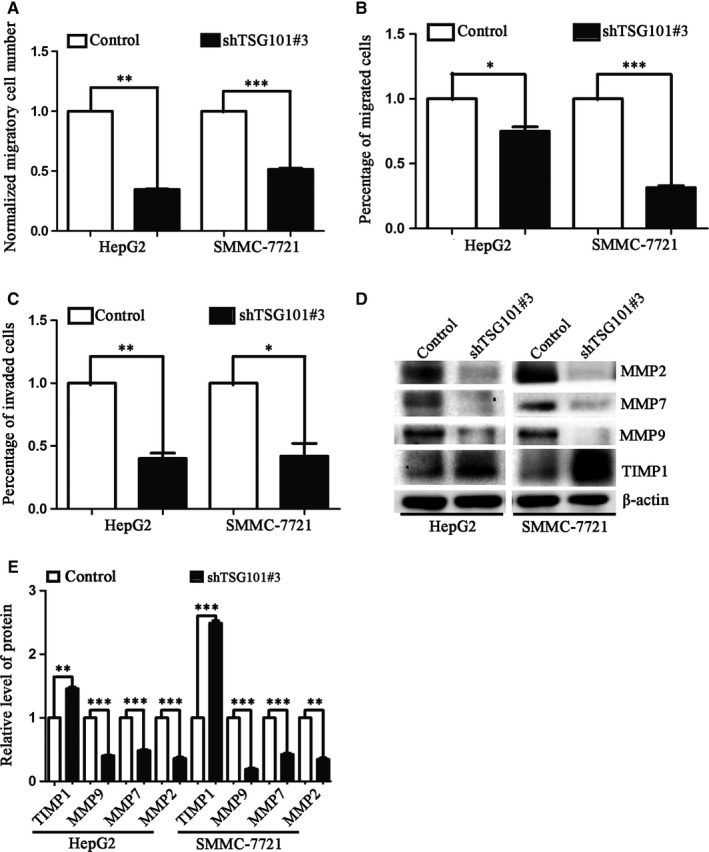
Down‐regulation of TSG101 inhibits HCC cell migration and invasion. A, Quantification of the wound healing assay performed in HepG2 and SMMC‐7721 cells upon knocking down of TSG101. B, Quantification of the transwell migration assay with TSG101 silenced HepG2 and SMMC‐7721 cells. C, Quantification of the transwell invasion assay with TSG101 silenced HepG2 and SMMC‐7721. D, Representative bolts and E, quantification to show the protein levels of TIMP1, MMP2, MMP7 and MMP9 in TSG101 silenced HepG2 and SMMC‐7721 cells. *P < 0.05, **P < 0.01, ***P < 0.001

To understand the clinical significance of TSG101, we analysed the correlation between TSG101 and HCC patient survival with TCGA database. It was showed that high levels of TSG101 were associated with poor prognosis in HCC patients (Figure 1A). Then, we took the advantage of loss‐of and gain‐of function approaches to determine the roles of TSG101 in the development and progression of human HCC. Firstly, we down‐regulated TSG101 expression by using its specific shRNA and observed its effects on cell proliferation, migration and invasion. To knock down TSG101, three shRNA targets (shTSG101#1, shTSG101#2 and shTSG101#3) were subcloned into a lentiviral vector pLV‐shRNA, a control shRNA served as a negative control. Then, we determined their silencing efficiency in suppressing TSG101 expression. As Figure 1B showed, the silencing efficiency of shTSG101#3 was approximately 80%. Therefore, we used shTSG101#3 and control to package the lentivirus in HEK293T cells for developing the stable cell lines with loss of TSG101. The cell lines were confirmed by GFP images (Figures S1A and B) and Western blotting (Figures S1C,D). To explore whether down‐regulation of TSG101 could affect the proliferation, migration and invasion of HCC cells, the behaviors of cell growth and motility were detected. The EdU incorporation assay showed that EdU‐positive cells in the TSG101 down‐regulated group reduced by 36.4% and 23.1% in HepG2 and SMMC‐7721 cells, respectively, comparing with the corresponding control (Figure S2A, Figure 1C). The ability of colony formation was also obviously suppressed by silencing of TSG101, comparing with the control group (Figure S2B, Figure 1D). In addition, the CCK‐8 assay showed the cell viability was reduced upon knocking down of TSG101 (Figure 1E,F). Correspondingly, cell cycle related proteins, like p53 and p21 were significantly increased after down‐regulation of TSG101 (Figure 1G,H). Next, we analysed the cell migration and invasion upon knocking down of TSG101. As shown in Figure S2C, Figure 2A, the wound healing assay indicated that the number of migratory cells was approximately decreased by 70% and 51% in HepG2 and SMMC‐7721 cell line, respectively. And the transwell migration assay (‐Matrigel) obtained similar results with the wound healing assay (Figure S1D, Figure 2B). In addition, the transwell invasion assay (+Matrigel) displayed that the number of invaded cells was approximately reduced by 62% and 59% compared with the control group, respectively (Figure 2C). Correspondingly, biomarkers of cell invasion and migration, like MMP2, MMP7 and MMP9 were significantly decreased and MMPs inhibitor TIMP1 (Tissue inhibitors of metalloproteinases 1) was increased after down‐regultion of TSG101 (Figures 2D,E). These results suggest that TSG101 is involved in the proliferation, migration and invasion of HCC cells and down‐regulation of TSG101 inhibits the proliferation, migration and invasion of HCC cells.

### 3.2 Overexpression of TSG101 promotes the proliferation, migration and invasion of HCC cells

**Figure 3:**
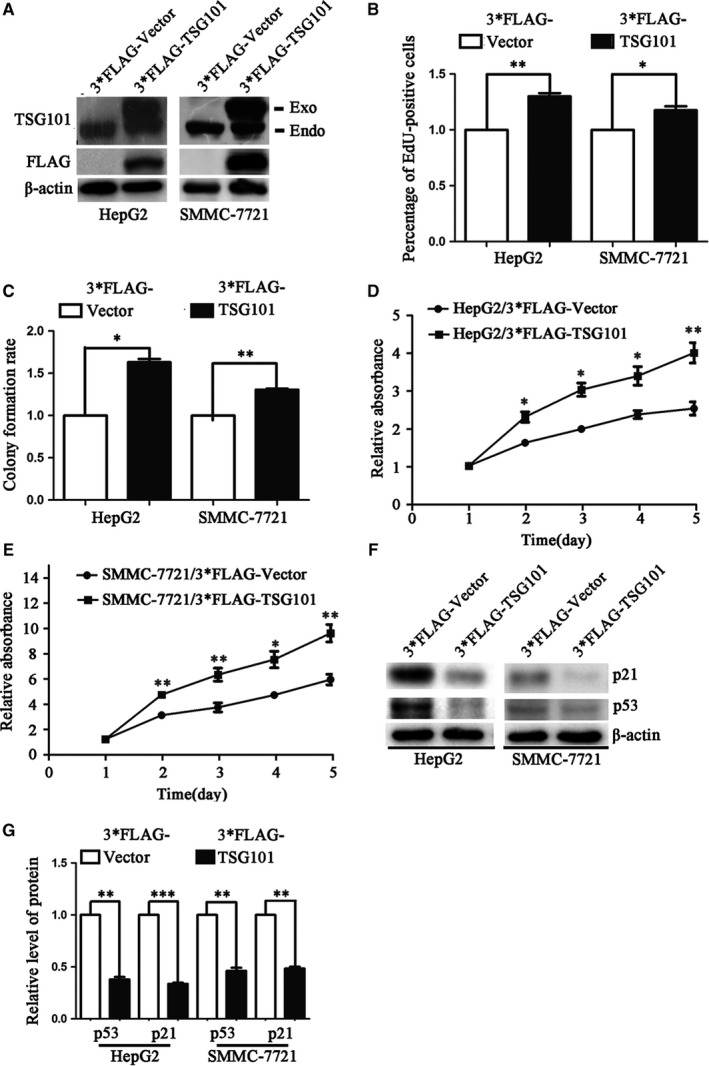
Overexpression of TSG101 promotes the proliferation of human HCC cells. A, The overexpressing efficiency of 3*FLAG‐TSG101 was verified by Western blotting with TSG101 antibody or FLAG antibody in HepG2 and SMMC‐7721 cells. B, Quantification of the EdU incorporation assay performed in HepG2 and SMMC‐7721 cells upon overexpressing of TSG101. C, Quantification of the colony formation assay with TSG101 overexpressed HepG2 and SMMC‐7721 cells. D and E, CCK8 assay results. F, Representative bolts and (G) quantification to show the protein levels of p53 and p21 in TSG101 overexpressed HepG2 and SMMC‐7721 cells. *P < 0.05, **P < 0.01, ***P < 0.001

**Figure 4:**
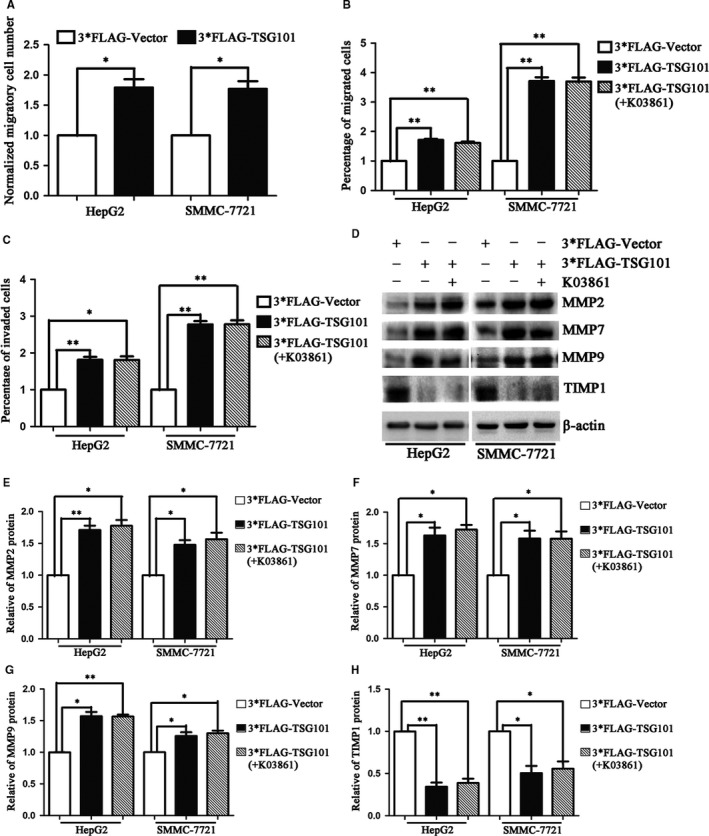
Overexpression of TSG101 promotes HCC cell migration and invasion. A, Quantification of the wound healing assay performed in HepG2 and SMMC‐7721 cells upon overexpressing of TSG101. B, Quantification of the transwell migration assay with TSG101 overexpressed HepG2 and SMMC‐7721 cells. C, Quantification of the transwell invasion assay with TSG101 overexpressed HepG2 and SMMC‐7721 cells. D, Representative bolts and E‐H, quantification to show the protein levels of p53 and p21 in TSG101 overexpressed HepG2 and SMMC‐7721 cells. *P < 0.05, **P < 0.01, ***P < 0.001

To further investigate the roles of TSG101 on the proliferation, migration and invasion of HCC cells, we then transiently transfected 3*FLAG tagged TSG101 cDNA into HepG2 and SMMC‐7721 cells to achieve the gain‐of function. The expression efficiency of TSG101 was confirmed by Western blotting (Figure 3A). Then, we asked whether the cell proliferation, migration and invasion were aggravated upon TSG101 overexpression. Therefore, 24 hours after transfection, the cells were used to evaluate the cell growth and motility. The EdU incorporation assay showed that, compared with the 3*FLAG group, EdU‐positive cells of the 3*FLAG‐TSG101 group increased by 34.2% and 24.6% in HepG2 and SMMC‐7721 cells, respectively (Figure S3A, Figure 3B). The ability of colony formation was also obviously increased upon transfecting with 3*FLAG‐TSG101, comparing with the 3*FLAG group (Figure S3B, Figure 3C). In addition, the CCK‐8 assay revealed that the proliferation rate of the 3*FLAG‐TSG101 group was significantly increased (Figure 3D,E). Correspondingly, p53 and p21 were significantly decreased after overexpression of TSG101 (Figure 3F,G). Subsequently, we examined the cell migration and invasion upon overexpression of TSG101. As shown in Figure S3C and Figure 4A, the wound healing assay indicated that, compared with the control group, the number of migratory cells was approximately increased by 77% and 75% HepG2 and SMMC‐7721 cells, respectively. To exclude the effect of cell proliferation induced by TSG101 overexpression on cell migration and invasion assays, we performed the transwell assays in the presence of a proliferation blocker, CDK2 inhibitor (K03861). The transwell migration assay (‐Matrigel) obtained similar results with the wound healing assay (Figure S3D, Figure 4B). In addition, the transwell invasion assay (+Matrigel) displayed that the number of invasive cells increased by 80% and 170% in HepG2 and SMMC‐7721 cells, respectively (Figure S3E, Figure 4C). Correspondingly, MMP2, MMP7 and MMP9 were significantly increased and TIMP1 was decreased after overexpression of TSG101 (Figure 4D‐H). These results further suggest that TSG101 is involved in the proliferation, migration and invasion of HCC cells and overexpression of TSG101 promotes the proliferation, migration and invasion of HCC cells.

### 3.3 TSG101 positively regulates PEG10 to promote the proliferation, migration and invasion of HCC cells

**Figure 5:**
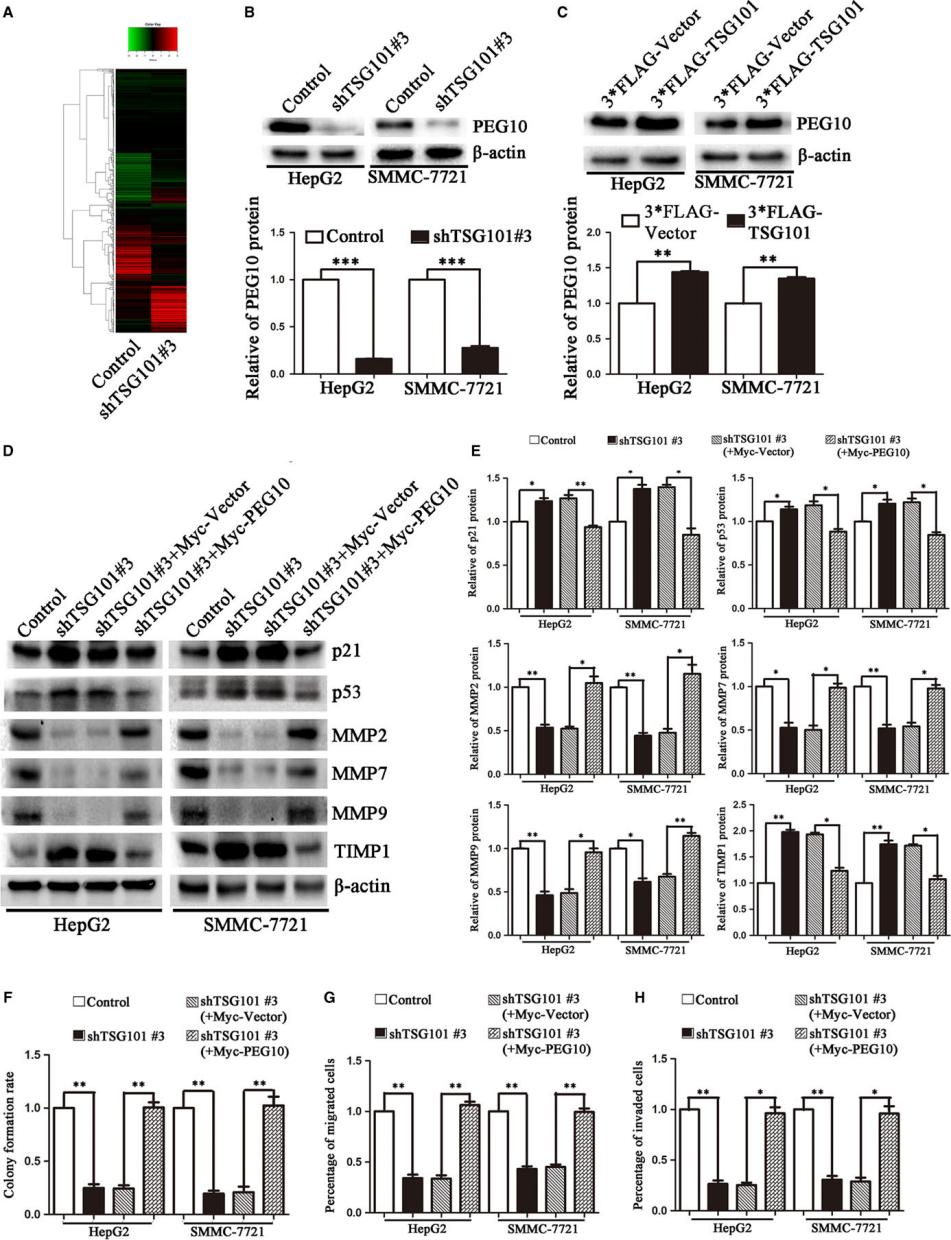
TSG101 positively regulates PEG10 in HCC cells. A, iTRAQ proteomics analysis to compare the protein expression between control and shTSG101#3 cells. B and C, Representative blots and quantification to show the protein level of PEG10 in TSG101 silenced or overexpressed HepG2 and SMMC‐7721 cells. D, Representative bolts and E, quantification showed overexpression of PEG10 effectively rescued the expressions of p21, p53, MMPs and TIMP1. F‐H, Colony formation assay, transwell migration and invasion assay showed overexpression of PEG10 effectively restored the cell proliferation, migration and invasion induced by knocking down of TSG101. *P < 0.05, **P < 0.01, ***P < 0.001

The above results have shown that TSG101 is important for the proliferation of HCC cells. However, the underlined mechanisms should be addressed. Thus, we performed an iTRAQ proteomics analysis to compare the protein expression between control and shTSG101#3 (Figure 5A). A total of 5400 peptides were detected in three independent biological replicates among four groups. Then, we analysed the identified peptides. As a result, there were 61 more than 2‐fold changed proteins were identified. Among the changed proteins, PEG10 has been demonstrated to promote cancer cell proliferation, migration and invasion in various human cancers,^24^, ^25^, ^26^, ^27^ and it has been reported to be involved in the regulation of the cell cycle related regulatory factors and the extracellular matrix metalloproteinases.^27^, ^28^, ^33^, ^34^ Thus, we has been suggested that whether PEG10 was involved in TSG101 regulated HCC cell proliferation, migration and invasion. Then, we confirmed the iTRAQ results by Western blotting. It was showed that silencing of TSG101 significantly decreased the expression of PEG10, while overexpression of TSG101 increased it (Figure 5B,C). To confirm TSG101 accelerates the proliferation, migration and invasion of HCC cells through regulating PEG10, we performed the rescue experiments by overexpressing Myc‐PEG10 in TSG101 down‐regulated cells. It was found that overexpression of PEG10 effectively rescued the expressions of p21, p53, MMPs and TIMP1 (Figure 5D,E), and also restored the cell proliferation, migration and invasion induced by knocking down of TSG101 (Figure 5F‐H). In conclusion, these results suggest that TSG101 positively regulates PEG10 thereby promoting the proliferation, migration and invasion of HCC cells.

### 3.4 TSG101 stabilizes PEG10 by protecting it from proteasomal degradation

**Figure 6:**
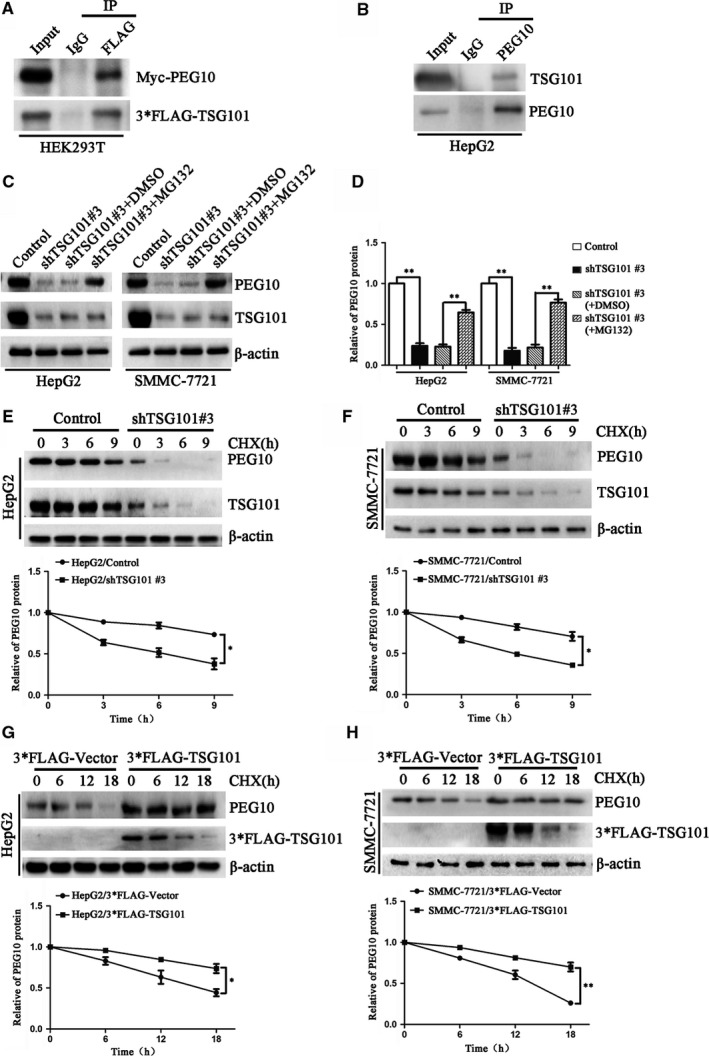
TSG101 interacts with and stabilizes PEG10 by protecting it from proteasomal degradation. A, Co‐immunoprecipitation assay showed that FLAG tagged TSG101 interacted with Myc tagged PEG10 in HEK293. B, Endogenous TSG101 and PEG10 formed into a complex in HepG2 cells. C, Representative bolts and D, quantification showed proteasome inhibitor MG‐132 could block the degradation of PEG10 in TSG101 down‐regulated cells. E and F, Representative bolts and quantification showed knock down of TSG101 decreased the stability of PEG10. G and H, Representative bolts and quantification showed overexpression of TSG101 increased the stability of PEG10. *P < 0.05, **P < 0.01, ***P < 0.001

To address how TSG101 regulates PEG10, we firstly explored the interaction between TSG101 and PEG10. It was found that FLAG tagged TSG101 interacted with Myc tagged PEG10 in HEK293 and HepG2 cells (Figure 6A,B). Also, the endogenous TSG101 and PEG10 formed into a complex in HepG2 cells (Figure 6C). Since it has been reported that TSG101 is frequently involved in protein ubiquitination and degradation, we thought whether TSG101 is involved in regulating the stability of PEG10. To answer this question, we treated the TSG101 silenced cells with proteasome inhibitor MG‐132, and found that MG‐132 could block the degradation of PEG10 in TSG101 down‐regulated cells (Figure 6D,E). Then, we treated the cells with cycloheximide (CHX), a protein synthesis inhibitor, and detected the stability of PEG10 upon knocking down or overexpressing of TSG101. It was showed that PEG10 was much more unstable upon knocking down of TSG101 in HepG2 and SMMC‐7721 cells (Figure 6F,G), however, the stability was significantly increased by overexpressing of TSG101 in HepG2 and SMMC‐7721 cells (Figure 6H,I).

### 3.5 TSG101 and PEG10 are up‐regulated and correlated in HCC patients

**Figure 7:**
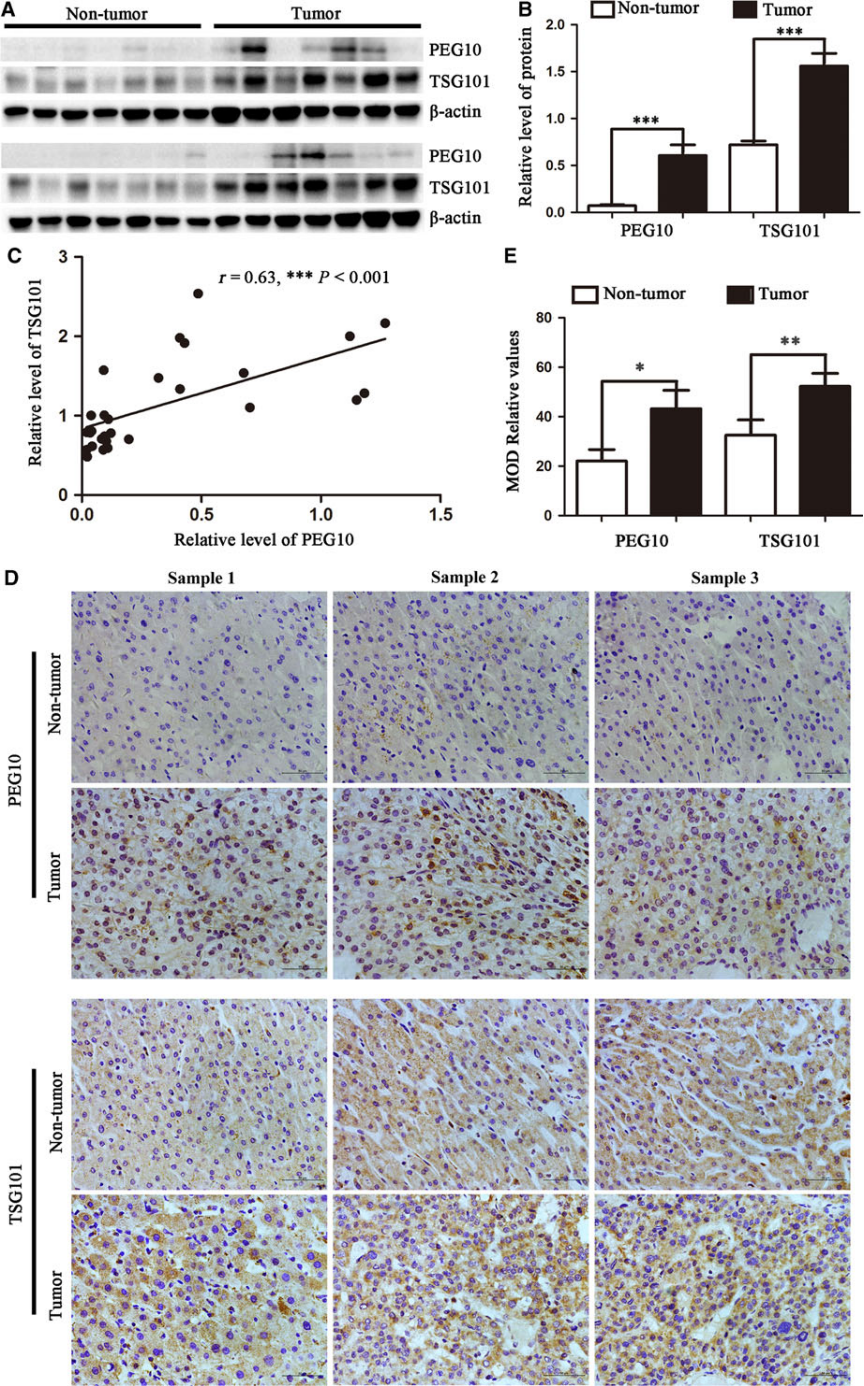
Expression of TSG101 and PEG10 in human HCC patients. A, Total proteins isolated from non‐tumor and HCC tissues were analyzed by Western blotting for assessment of TSG101 and PEG10. B, Statistical chart showed the expression levels of TSG101 and PEG10 in non‐tumor and HCC tissues. The ratios indicate the levels of TSG101 and PEG10 to β‐actin levels with respect to each sample. C, The correlation of TSG101 expression with PEG10 in non‐tumor and HCC tissues. *r* = 0.63; *P *< 0.001. D, Representative images of TSG101 and PEG10 from non‐tumor and HCC tissues determined by immunohistochemistry, Bar = 50 μm. E, The histogram showed the quantitative analysis of the relative level of TSG101 and PEG10. * *P *<* *0.05, ** *P *<* *0.01, *** *P *<* *0.001

Finally, we studied the clinical relevance of TSG101 and PEG10 and their relationship in clinical HCC patients. As is shown in Figures 7A,B, the protein level of TSG101 and PEG10 in HCC samples was approximately 3‐6 folds higher than those of non‐tumour tissues. Next, we analyzed the correlation between TSG101 and PEG10 and found that there was a direct correlation between TSG101 and PEG10 levels in non‐tumour tissues and HCC patients (*r* = 0.63, *P *<* *0.001; Figure 7C). In addition, we assessed the expression and distribution of TSG101 and PEG10 in non‐tumour tissues and HCC patients by immunohistochemistry. Similarly, the expression of TSG101 and PEG10 in HCC tissues were significantly increased compared with the non‐tumour tissues, which was in line with our results found above (Figure 7D,E). Collectively, these results suggest that TSG101 and PEG10 are up‐regulated in human HCC patients and further confirm the positive correlation between TSG101 and PEG10 levels.

## 4 DISCUSSION

TSG101 is a cancer‐related protein that has been reported to play important roles in many cellular processes, including endosomal trafficking, ubiquitination, virus budding and cell survival.^5^, ^6^, ^7^, ^8^, ^9^ Recently, it is found that the expression of TSG101 is frequently up‐regulated in human ovarian cancer, colorectal carcinoma, papillary thyroid carcinoma, gastrointestinal tumour, and gallbladder cancer,^16^, ^17^, ^18^, ^19^, ^20^ which indicates that TSG101 could be a potential biomarker in human cancers. However, the expression pattern in human HCC has not yet been clarified. In our present study, we detected the protein expression of TSG101 in HCC tissues and non‐tumour liver tissues. We discovered that TSG101 protein was expressed increasingly in the patients with HCC, comparing with the non‐tumour liver tissues. We also analyzed the effects of TSG101 on the proliferation, migration and invasion of HCC cells and found that down‐regulating of TSG101 inhibited the cell proliferation, migration and invasion, whereas overexpression of TSG101 promoted them. These results suggest that TSG101 exerts a tumour‐promoting role in HCC and might be acted as a potential biomarker for HCC.

It is well known that depression of specific tumour suppressor genes or activation of specific oncogenes is a key event for the development and malignant progression of HCC.^31^, ^35^, ^36^, ^37^ This usually includes, on the one hand, any gain‐of function mutation or up‐regulation of the upstream activators, and, on the other hand, any loss‐of function mutation or down‐regulation of the upstream inhibitors. TSG101 has been shown to regulate the Akt and ERK proteins.^21^, ^22^ In this study, we have provided the evidence that TSG101 is involved in the regulation of another oncogenic protein, PEG10. We showed that TSG101 and PEG10 forms into a complex in HCC cells, and TSG101 could increase the protein stability of PEG10 by protecting it from proteasomal degradation, thereby promoting the proliferation, migration and invasion of HCC cells through regulating the expression of cell cycle related regulatory factors and the MMPs. Interestingly, it was also found that both TSG101 and PEG10 proteins are up‐regulated in HCC patients. Moreover, there was a direct correlation between TSG101 and PEG10 protein levels. In this case, we conclude that abnormal expression of TSG101 in human HCC patients will cause the up‐regulation of PEG10, which will facilitate the proliferation, migration and invasion of HCC. Therefore, up‐regulation of TSG101 may be an important event in the development and malignant progression of HCC.

In conclusion, we have firstly supplied the evidences linking TSG101 to the proliferation and malignant progression of human HCC through regulating oncogenic protein PEG10. These findings have provided some basis for further investigation of TSG101‐mediated signaling pathway and for evaluating the prognostic by analyzing TSG101 status in patients diagnosed as HCC. However, more profound explorations are needed to clarify the precise mechanisms of TSG101‐mediated PEG10 up‐regulation in human HCC.

## CONFLICT OF INTEREST

None.
