# Supplementary figures and images for: TSG101 promotes the proliferation, migration and invasion of hepatocellular carcinoma cells by regulating the PEG10
Source: J Cell Mol Med. 2018 Nov 18;23(1):70–82. doi: 10.1111/jcmm.13878 (PMC6307771; doi:10.1111/jcmm.13878)

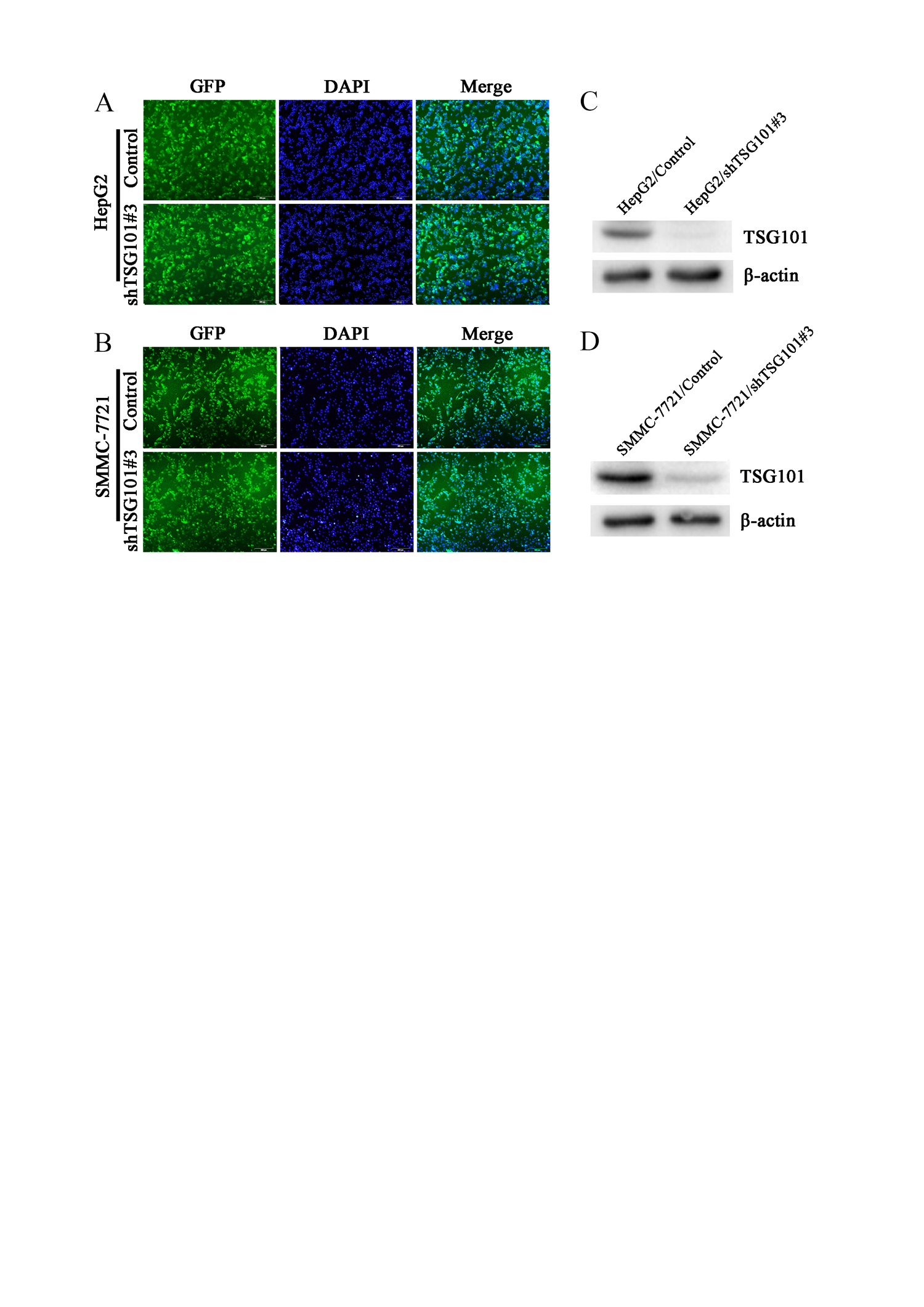

Supplement: Supplementary file 1 [file JCMM-23-70-s001.tif]

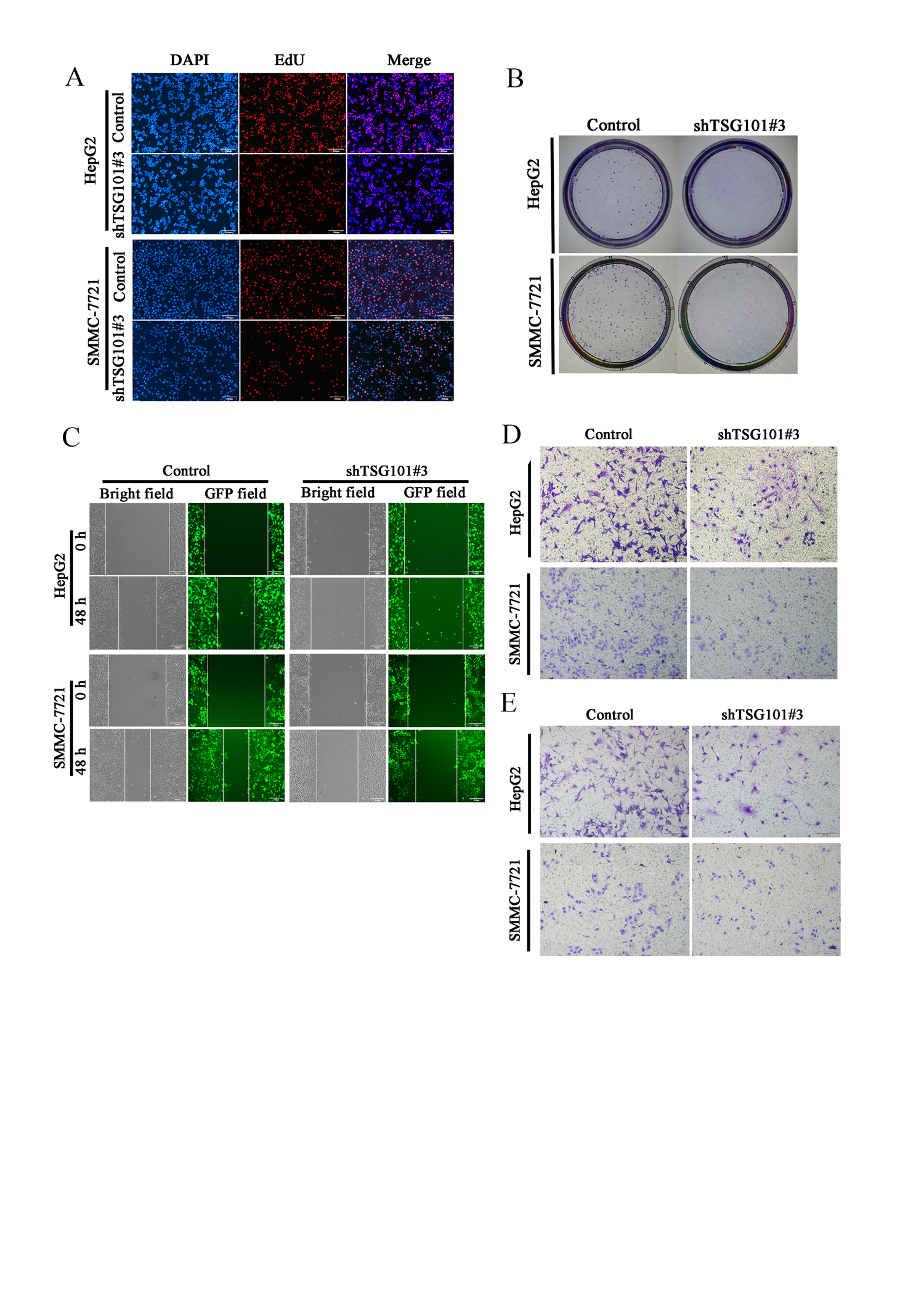

Supplement: Supplementary file 2 [file JCMM-23-70-s002.tif]

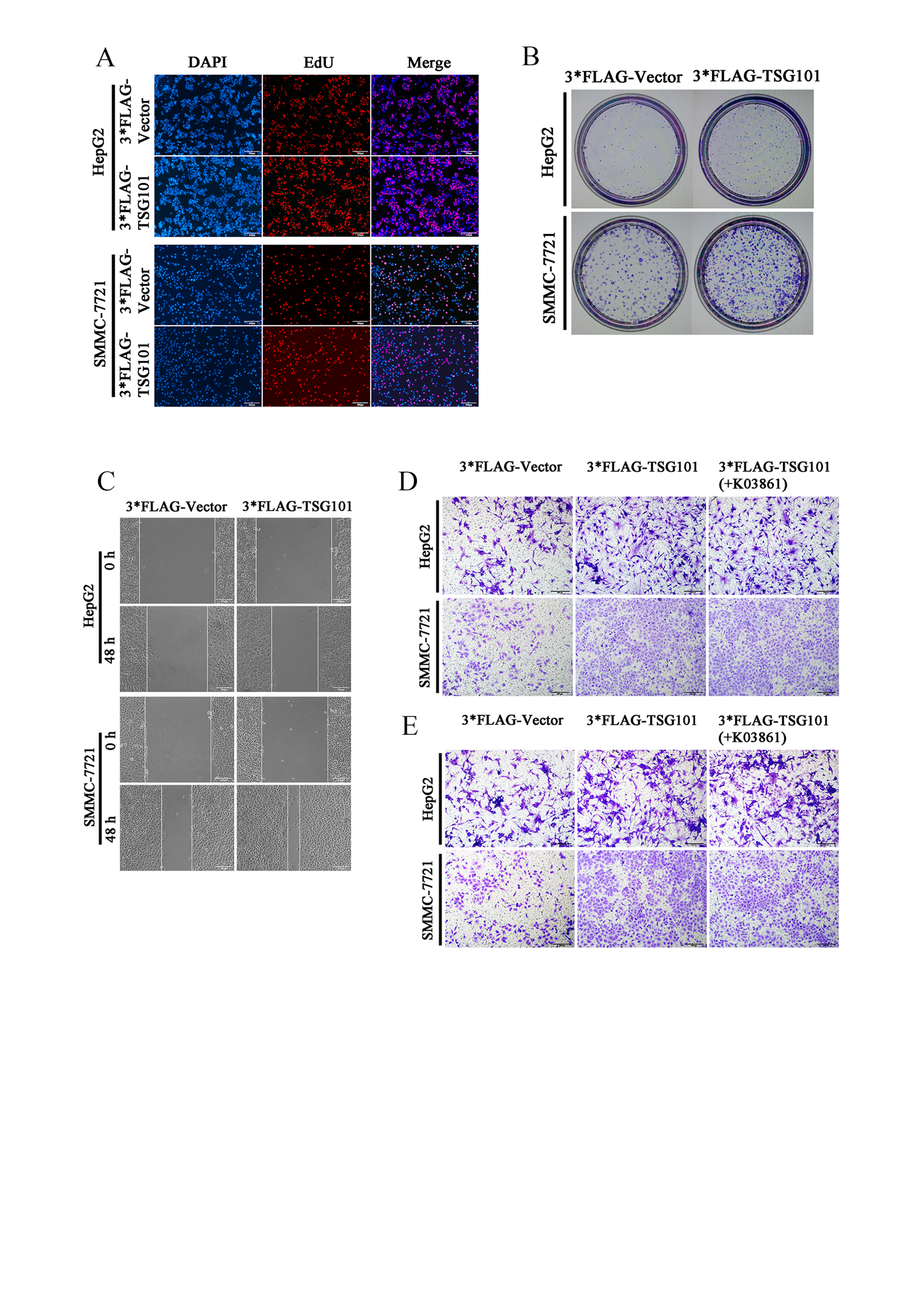

Supplement: Supplementary file 3 [file JCMM-23-70-s003.tif]
